# Non-Genetic Determinants of Mosquito Competence for Malaria Parasites

**DOI:** 10.1371/journal.ppat.1003365

**Published:** 2013-06-20

**Authors:** Thierry Lefèvre, Amélie Vantaux, Kounbobr R. Dabiré, Karine Mouline, Anna Cohuet

**Affiliations:** 1 MIVEGEC (Maladies Infectieuses et Vecteurs: Ecologie, Génétique, Evolution et Contrôle), UMR Universités Montpellier 1 & 2, CNRS 5290, IRD 224, Montpellier, France; 2 Institut de Recherche en Sciences de la Santé, Bobo Dioulasso, Burkina Faso; 3 Centre Muraz, Bobo Dioulasso, Burkina Faso; International Centre for Genetic Engineering and Biotechnology, India

## Abstract

Understanding how mosquito vectors and malaria parasites interact is of fundamental interest, and it also offers novel perspectives for disease control. Both the genetic and environmental contexts are known to affect the ability of mosquitoes to support malaria development and transmission, i.e., vector competence. Although the role of environment has long been recognized, much work has focused on host and parasite genetic effects. However, the last few years have seen a surge of studies revealing a great diversity of ways in which non-genetic factors can interfere with mosquito-*Plasmodium* interactions. Here, we review the current evidence for such environmentally mediated effects, including ambient temperature, mosquito diet, microbial gut flora, and infection history, and we identify additional factors previously overlooked in mosquito-*Plasmodium* interactions. We also discuss epidemiological implications, and the evolutionary consequences for vector immunity and parasite transmission strategies. Finally, we propose directions for further research and argue that an improved knowledge of non-genetic influences on mosquito-*Plasmodium* interactions could aid in implementing conventional malaria control measures and contribute to the design of novel strategies.

## Introduction

Despite ongoing control efforts, malaria remains one of the most devastating infectious diseases worldwide. The evolution of insecticide-resistant mosquito vectors and drug-resistant parasites, coupled with the current lack of adequate vaccines, has urged the scientific community to develop novel strategies for malaria control. One of the most promising approaches targets the mosquito vectors to disrupt parasite transmission [1]–[3]. As part of this effort, an important question is what determines mosquito vector competence for malaria parasites (**Box 1**)? In other words, why are some mosquitoes able to resist infection while others remain susceptible, thereby ensuring continued disease transmission?

**Box 1. Defining and measuring mosquito vector competence for malaria parasites**

The successful transmission of malaria parasites between humans requires a series of complex developmental transformations inside the mosquito vector. Shortly after the ingestion of an infectious blood meal, *Plasmodium* male and female gametes fuse to form zygotes within the mosquito midgut. Zygotes then develop into motile ookinetes that penetrate the gut wall to form oocysts. There, oocysts undergo several mitotic divisions resulting in hundreds of sporozoites that are released into the haemoceol 8–22 days post-infection (depending on the *Plasmodium spp.)*. At this stage, the parasites migrate to the salivary glands from which they can be injected into another vertebrate host during a subsequent blood meal.

Each step of this cycle is critical and the parasite commonly suffers massive reduction in population size during these transitions. Of the several hundred species of mosquitoes worldwide, only about 60 are known to be competent for malaria parasites, that is, they support the completion of each parasite developmental stage, from the gametes fusion and oocysts formation to the invasion of salivary glands and sporozoites transmission. Vector competence is a combined estimate of parasite infectivity and vector susceptibility and thus encompasses both host resistance mechanisms used to fight the infection and parasite infective mechanisms used to overcome the host's defenses. The degree of vector competence for malaria varies greatly between different mosquito species, and even between individuals from the same species or strain.

Vector competence can be measured in the laboratory using a number of experimental feeding assays. Mosquitoes can be exposed to a given dose of parasite gametocytes during blood-feeding on an infected vertebrate host (Direct Feeding Assay), or through a membrane containing either cultured parasites (Standard Membrane Feeding Assays) or blood drawn from naturally infected patients (Direct Membrane Feeding Assays). Each of these approaches measure infection-related traits, which characterize the success or failure of the infection and hence, vector competence. These traits are:

- *Parasite prevalence*. This is the proportion of malaria-exposed mosquitoes harboring at least one oocyst in their midgut (oocyst prevalence) or sporozoite in their salivary gland (sporozoite prevalence). A low prevalence indicates high anti-infection (i.e., qualitative) resistance to the parasite's establishment in the mosquitoes and/or low parasite infectivity.
- *Parasite intensity*. This is the number of oocysts in the guts, or the number of sporozoites in the salivary glands, of infected mosquitoes. A low intensity indicates high antigrowth (i.e., quantitative) resistance to parasite proliferation in the mosquitoes and/or low parasite development ability.

**Figure 1:**
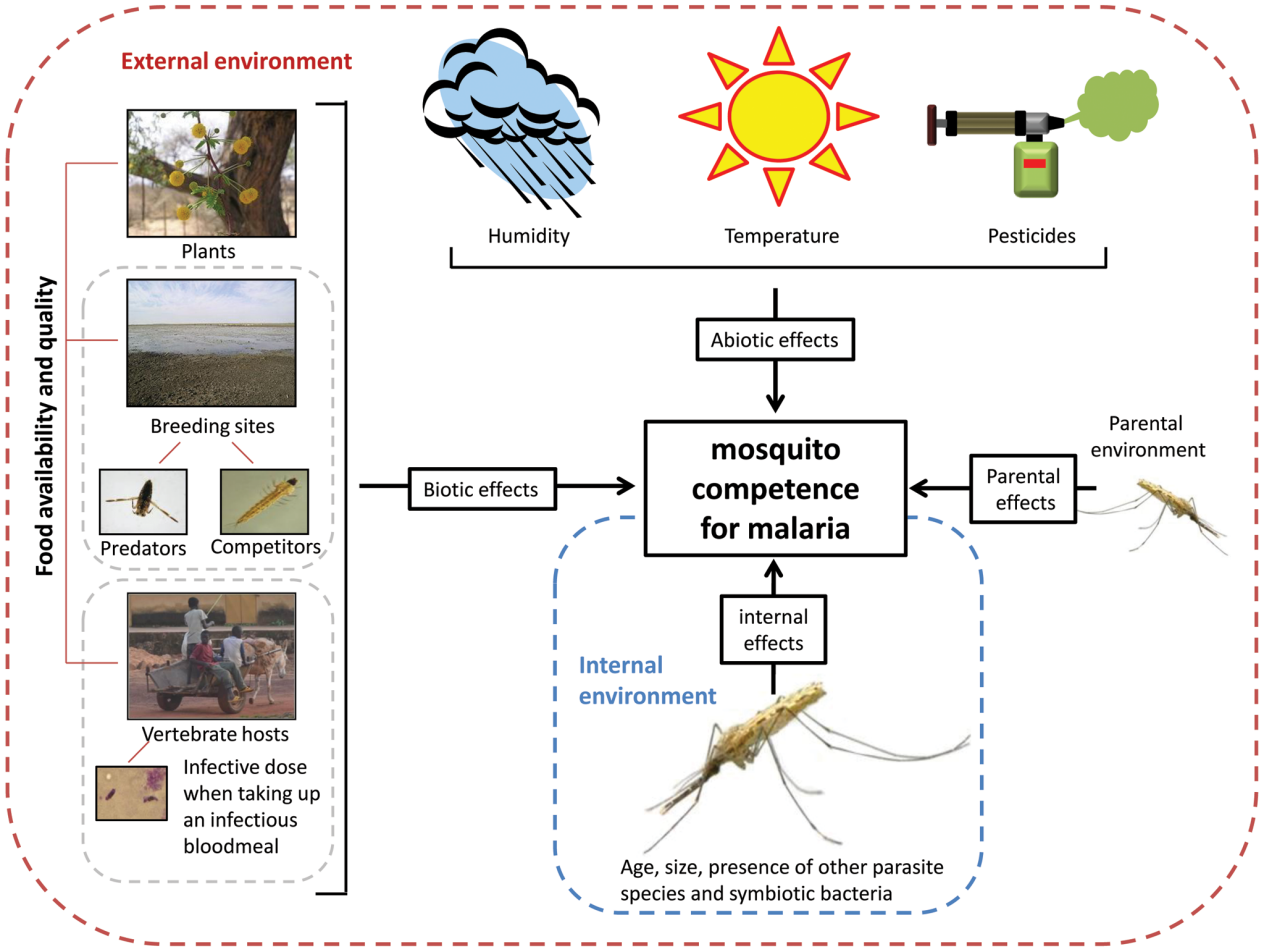
Diversity of ways in which non-genetic factors may influence mosquito competence for malaria parasites. The net effect of these factors on vector competence will depend on how they affect both the parasite's ability to establish and develop in the insect host and the mosquito's ability to resist and/or tolerate the infection. *All pictures downloaded from Wikipedia except the photo illustrating the vertebrate hosts (credit: Thierry Lefèvre) and the mosquito (credit: Nil Rahola).*

With these definitions in mind, there are numerous non-genetic factors that may influence vector competence via positive or negative effects on the parasite infectivity, development and virulence, as well as mosquito resistance and tolerance (**Figure 1**).

To address this question, intensive genetic, molecular, cellular, and physiological studies have been undertaken in recent years to investigate the mosquito immune response to malaria. These studies have revealed a number of mechanisms involved in preventing or limiting parasite development, including the Toll, IMD, Jak-Stat, and complement-like pathways [4]–[10]. Such findings have sparked interest in malaria control through the production of refractory transgenic mosquitoes [11], [12].

Although these studies generated a significant body of knowledge on the genetic and molecular mechanisms underlying mosquito competence to malaria parasites, they mostly used unnatural laboratory host-parasite associations, which can be poor approximations of what occurs in natural ecosystems [13]–[15]. Bearing in mind this criticism, recent studies have focused on natural vector-parasite interactions and confirmed that mosquito genetics play a major role in determining vector competence [16], [17]. These studies have also uncovered complex vector-parasite genetic interactions, whereby the outcome of infection depends on specific interactions between vector and parasite genotypes (see **Glossary, File S1**) [16], [18], [19].

In addition to genetic factors, the rapidly growing field of ecological immunology [20] demonstrates that abiotic and biotic factors can also play critical roles in modulating host-parasite interactions [21], [22]. Despite its medical relevance and the long recognition of temperature effect on *Plasmodium* development [23], [24], ecological immunology of mosquito-*Plasmodium* interactions is still in its infancy [14].

The primary scope of this review is to provide an up-to-date appraisal on the various ways in which these non-genetic factors influence vector competence for malaria parasites. We show that factors such as temperature, mosquito larval and adult diets, and microbial gut flora can play a major role, comparable to host and parasite genotypes, in shaping mosquito competence. We also stress that non-genetic-mediated competence can have important evolutionary and epidemiological implications. Finally, we argue that an improved understanding of the interactions between mosquitoes, the malaria parasites they transmit, and the environment is essential if we are to develop control measures that are efficient under real world conditions.

## Non-Genetic Influences on Mosquito Competence

In their natural habitats, mosquitoes are constantly challenged with various biotic and abiotic pressures, including resource limitation, competition, predation, temperature variations, and pesticides, which affect their reproduction and survival. Each of these factors may also affect their competence as vectors of *Plasmodium* (**Figure 1**). Most studies to date have focused on the effects of temperature and gut microbial flora, but other factors such as larval and adult diet, infection history, and maternal environment can also play a role in determining the outcome of mosquito infection. In the vast majority of cases however, the underlying mechanisms through which these non-genetic factors affect vector competence (direct effects on the parasite versus indirect effects via the mosquito immune defenses) remain elusive. Non-genetic factors most likely affect competence through a complex mediation of mosquito immunity and *Plasmodium* development.

### Temperature

Mosquitoes are small, ectothermic insects whose life-history traits, including larval development, adult survival, and immune response, strongly depend on ambient temperature [25]–[28]. Likewise, the development of many mosquito-borne pathogens is known to be temperature sensitive [27]. In particular, extensive early research identified that the permissive temperatures for *Plasmodium* sporogonic development range from 16°C to 35°C (**Table 1**) [23], [24], [29]–[31]. These early studies also showed that parasites develop faster at higher temperatures, suggesting that malaria transmission would be more intense under warmer conditions [24], [31]–[34]. However, experiments performed within the permissive temperature range indicate that, while mosquitoes do become infectious more quickly as temperature rises, their competence decreases (around 30°C in *Anopheles gambiae-Plasmodium falciparum* [35], 21–24°C in *Anopheles quadrimaculatus/Anopheles stephensi-Plasmodium berghei* [34], and 24–26°C in *An. stephensi-Plasmodium yoelli* [36]).

**Table 1:** Existing evidence for non-genetic influences on mosquito competence for malaria parasites.

Factor	Effect and magnitude	Biological system	Refs
**Temperature**	Parasite development rate increased with temperature until a threshold was reached, at which point parasite survival sharply decreased.	*An. stephensi - P. falciparum* *An. stephensi* - *P. falciparum*	[32] [33]
	Within the parasite thermal limit, high temperatures accelerated parasite development but decreased vector competence. A change from 22°C to 26°C resulted in a five-fold decrease in sporozoite prevalence of *P. yoeli–*infected *An. stephensi*.	*An. stephensi* - *P. yoelii* *An. quadrimaculatus & An. stephensi* - *P. berghei*	[34] [36]
	Within the parasite thermal limit, high temperatures decreased vector competence. Temperature increases from 27°C to 30°C and 32°C reduced oocyst prevalence from 15.9% to 8.5% and 6.4%.	*An. gambiae* - *P. falciparum*	[35]
	Compared to an equivalent constant mean temperature, competence increased when diurnal fluctuations occurred around low mean temperatures (from about 0% to 10% sporozoites prevalence at 16°C) but decreased with fluctuations around high mean temperatures (from 30% to about 0% sporozoites prevalence at 26°C).	*An. stephensi* - *P. chabaudi*	[37]
	Mosquito ability to melanize foreign entities declined with increasing temperatures. The percentage of melanized beads dropped from 63% to 53% and 30% with temperature increases from 24°C to 27°C and 30°C.	*An. gambiae -* Sephadex beads	[28]
	Mosquito immune responses showed complex interactions with temperature, time, and nature of immune challenge.	*An. stephensi* - Sephadex beads, fluorospheres, bacteria	[26]
**Food**	Glucose-deprived females displayed greater competence than females fed on glucose *ad libitum* (i.e., they harbored about twice as many oocysts).	*An. stephensi - P. chabaudi*	[43]
	Females fed on 4% glucose displayed greater competence than females fed on 2% and 6% glucose (i.e., they harbored about twice as many oocysts).	*An. stephensi* - *P. yoelii yoelii*	[42]
	The melanization response to foreign entities showed a two-fold increase with increasing sugar concentration following a blood meal.	*An. stephensi -* Sephadex beads	[46]
	Nutritional deprivation during the larval stages decreased melanization response (i.e., melanization decreased by three-fold with a four-fold decrease in larval food quantity).	*An. gambiae* - Sephadex beads	[28]
	Greater competence in females fed double blood meals compared to single blood meals (i.e., 35% oocyst prevalence on double blood meals compared to 25% on single blood meals).	*An. gambiae - P. falciparum*	[44]
**Gut microbiota**	High bacterial load and diversity decreased competence (i.e., aseptic mosquitoes harbored about 8 times more oocysts than their septic counterparts).	*An. gambiae - P. falciparum & P. berghei*	[50]–[52]
	A specific bacterial isolate conferred total refractoriness.	*An. gambiae - P. berghei & P. falciparum*	[51]
	Field-collected infected mosquitoes harbored about 2.5 times more enterobacteria than uninfected mosquitoes.	*An. gambiae - P. falciparum*	[53]
**Infection history**	Co-infection with entomopathogenic fungi decreased competence (i.e., 35% sporozoite prevalence in malaria-infected mosquitoes compared to 8% in co-infected mosquitoes).	*An. stephensi - P. chabaudi & Metarhizium anisopliae* & *Beauveria bassiana*	[54]
	Co-infection with microsporidian parasites decreased competence (i.e., 58.5% oocyst prevalence with a mean number of 8.9 oocysts in microsporidian-infected mosquitoes compared to 81.8% and 20.7 in microsporidian-uninfected mosquitoes).	*An. gambiae - P. berghei & Vavraia culicis &* Sephadex beads	[56]
	Co-infection with filarial worms decreased competence (i.e., about four-fold and 50% decrease in oocyst intensity and prevalence, respectively).	*Armigeres subalbatus & Ae. aegypti - P. gallinaceum, Brugia malayi, B. pahangi & Dirofilaria immitis*	[55]
	Co-infection with two malaria parasite species decreased competence by two-fold for one of the two malaria species.	*Ae. aegypti - P. gallinaceum & P. juxtanucleare*	[58]
	Previous malaria infection decreased by three-fold the competence to a subsequent malaria infection.	*An. gambiae - P. falciparum & P. berghei*	[59]
**Maternal effects**	Infection with microsporidian parasites decreased competence in the offspring (i.e., 70% of the offspring of microsporidian-free mothers infected with *P. berghei* against 42% of *V. culicis*–infected females). Food deprivation increased the likelihood of infection in the offspring by 32%.	*An. gambiae - P. berghei*	[61]
	Offspring from mothers inoculated with foreign entities had a similar melanization response than offspring from unchallenged mothers.	*Ae. aegypti* - Sephadex beads	[60]
**Mosquito age**	The percentage of melanized beads decreased from 50% in <1-day-old females to about 10% in >1-day-old females.	*An. gambiae* - Sephadex beads	[64]
	No age effect on mosquito susceptibility to entomopathogenic fungi.	*An. gambiae* - *Metarhizium anisopliae* & *Beauveria bassiana*	[65]
	No age effect on competence for malaria parasites.	*An. gambiae - P. falciparum*	[44]
**Mosquito body size**	Melanization response was stronger in large than in small females.	*An. gambiae* - Sephadex beads	[28]
	Competence increased with size.	*An. gambiae - P. falciparum* *An. dirus - P. falciparum*	[73], [74]

These results suggest that malaria transmission may fall, rather than rise, at higher temperatures. However, they must be interpreted with caution as mosquitoes do not experience constant temperature regimens in nature. Paaijmans et al. [37] demonstrated that *An. stephensi* competence for *Plasmodium chabaudi* can be influenced by temperature fluctuations in complex ways depending on the mean temperature around which the fluctuation occurs. In particular, compared to an equivalent constant temperature, competence increased when diurnal fluctuations occurred around low mean temperatures but decreased with fluctuations around high mean temperatures [37]. Furthermore, mosquitoes are able to dampen temperature extremes through behavioral thermoregulation. The standard estimates of ambient outdoor temperature used in the experiments described above may therefore erroneously reflect the conditions that mosquitoes and parasites experience in the field [38].

### Diet

Food availability and quality has repeatedly been shown to be an important environmental factor in relation to insect host immunity and infection [39]. Diet influences on infections can be mediated either through toxic secondary metabolites [40] or differences in nutritional value that, in turn, affect host immunity [41]. Studies on diet effects in mosquito-*Plasmodium* interactions have lagged behind, but a handful of findings indicate that nutrition can influence mosquito competence [28], [42]–[46]. These studies have proven difficult to reconcile however, as the competence and immune response of food-deprived larvae or adult mosquitoes was reported to be higher in some instances [28], [43], [46] but lower in others [42], [44] (**Table 1**). It remains unclear whether these discrepancies are due to species-specific differences or to the type and degree of nutritional stress.

**Figure 2:**
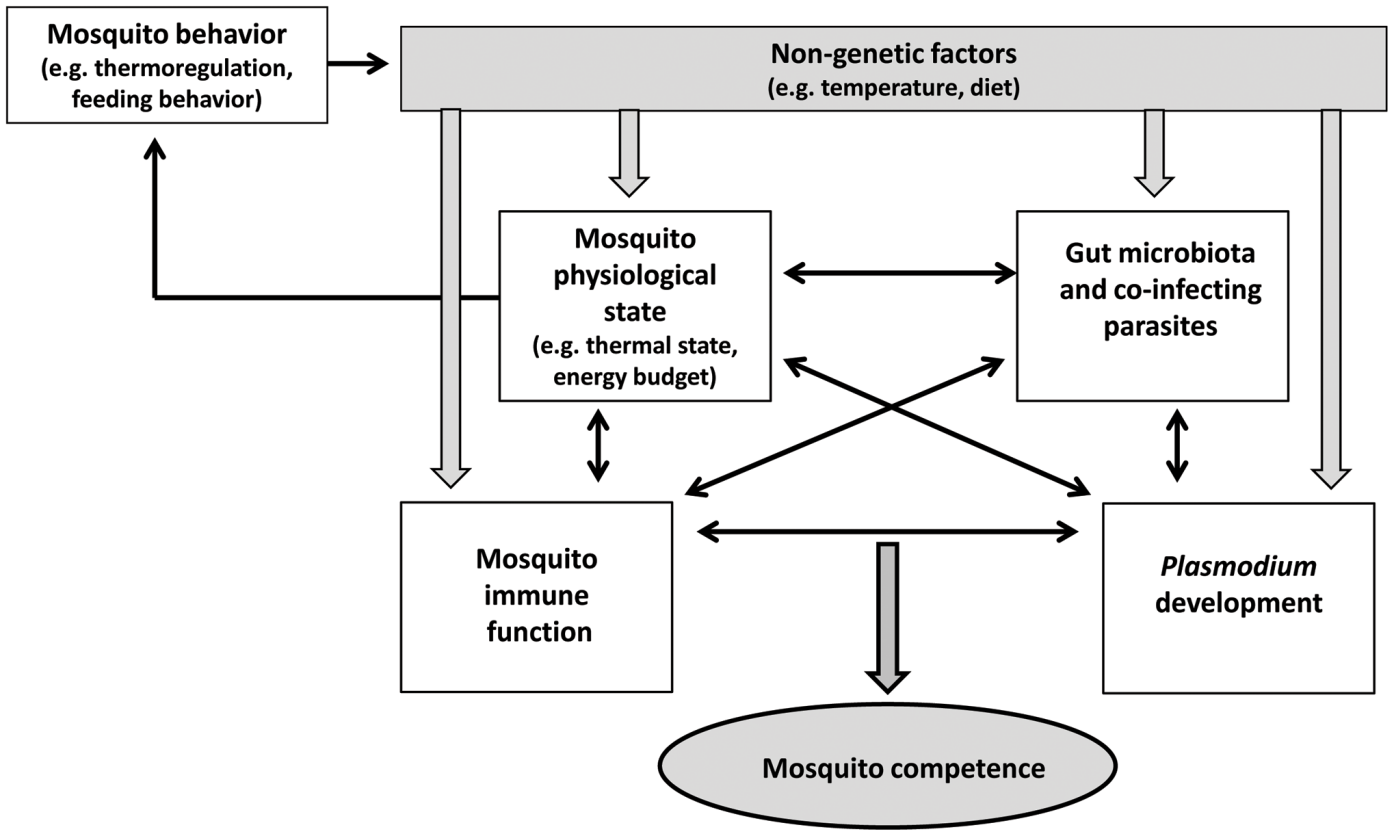
Complex environmental mediation of mosquito competence for malaria parasites. Environmental factors such as temperature and diet can affect parasite development and mosquito physiological state and immune response, which can all interact with microbial symbionts and other co-infecting parasites. Because physiological feedbacks modulate behavior, the potential exists for the mosquito to adjust some environmental conditions (diet, temperature) to optimize its microbial interactions and immune function and to increase its resistance to infection. Alternatively, malaria parasites might manipulate host behaviors (e.g., feeding, thermal behavior) to their own benefit. Modified from Ponton et al. [47].

The relationships between nutrition, immunity, and infection are complex [47]. For example, nutrition may indirectly influence *Plasmodium* development by mediating changes in the gut microbiota, such as proposed for other host-parasite interactions (**Figure 2**, [47]). Furthermore, mosquitoes can access a large variety of nutritive resources that will then be allocated to functional traits such as immune functions. Until now, most studies have focused on quantitative, as opposed to qualitative, diet changes, and have used non-natural food sources (e.g., glucose solutions or blood from inappropriate vertebrate hosts). This is unfortunate, as female mosquitoes readily ingest carbohydrates from a wide range of plant species in addition to blood meals [48]. Whether natural plant diversity affects mosquito competence for malaria through toxic secondary metabolites and/or nectar nutritional properties remains to be discovered.

### Gut Microbiota, Infection History, and Immune Priming

Recently, there has been a renewed interest in the role of mosquito bacterial gut communities on vector competence [49]. Both bacterial abundance and diversity appear to play a role, with high bacterial loads and specific bacterial isolates associated with compromised *Plasmodium* early development [50]–[52] (**Table 1**). For example, Cirimotich et al. [51] identified an enterobacterium isolated from wild mosquitoes that confers total refractoriness to *P. falciparum* through the production of reactive oxygen molecules. However, the mechanisms responsible for bacteria-mediated reduction in vector competence can also be indirect. In this case, the bacteria seem to impede the development of malaria by priming the mosquito immune system [50], [52] (**Figure 2**).

It is worth noting that the presence and abundance of gut bacteria is not always associated with reduced competence. For example, a recent study found that enterobacteria abundance was higher in field-collected *P. falciparum*–infected mosquitoes than in their uninfected counterparts, suggesting that some bacteria may promote parasite development [53]. However, it was unclear whether these enterobacteria indeed had an effect on parasite development or whether their increased abundance was a mere consequence of *Plasmodium* presence, or of the mosquito's general vulnerability to both enterobacteria and malaria parasites. Overall, our understanding of microbiota-mosquito-*Plasmodium* interactions is still limited, and more studies are required to determine the extent to which natural resident microbial flora of vectors contributes to competence for malaria parasites.

In addition to their gut bacteria, mosquitoes harbor a wide range of organisms which may also impact *Plasmodium* development. For example, recent studies show that microsporidian, fungal, and filarial parasites can reduce concomitant *Plasmodium* infections [54]–[56] (**Table 1**). Co-infection by multiple species of malaria parasites has also been reported [57], but the consequences for vector competence are largely unknown. In one experiment, simultaneous infection with two species of avian malaria parasite resulted in a decreased competence for one of the co-infecting parasite species, possibly due to direct interspecific negative interference during fertilization [58].

Consecutive infections, where an already infected mosquito is exposed to a second malaria parasite strain or species, can be frequent under natural conditions and can impact vector competence [57]. For example, mosquitoes experiencing a first malaria infection display enhanced immunity to a subsequent and similar parasite challenge [59]. Interestingly, the resulting reduction in secondary infection was only observed in mosquitoes harboring midgut bacteria—not in aseptic counterparts—suggesting that mosquito immune priming was mediated by gut microbiota [59].

Finally, previous exposure to parasites may not only confer lasting protection within an individual but may also extend to the next generation. Evidence for such trans-generational immune priming is becoming common in the field of insect ecological immunity [20], yet we are aware of only two studies that have addressed this possibility specifically in mosquitoes. The first study found no support for trans-generational immune priming, with female *Aedes aegypti* from mothers inoculated with foreign entities having a similar melanization response as females from unchallenged control mothers [60]. The second study, however, showed that female *An. gambiae* from mothers infected with a microsporidian parasite displayed lower competence for *P. berghei* than females from uninfected mothers [61]. Thus, microsporidian parasites may limit malaria infection not only within an individual [56] but also in its offspring [61]. These results suggest that parental environment can have important consequences on offspring competence for malaria parasites. However, more studies are required to test the extent to which such parental effects can be generalized across a range of mosquito-*Plasmodium* combinations and parental environmental conditions.

### Mosquito Intrinsic Factors

Under field situations, mosquito populations are made up of individuals that differ not only in their genetic background, but also in regard to factors such as age, reproductive status, and body size. This heterogeneity can have important consequences on vector competence. For example, the host immune system may weaken with age, resulting in an increased susceptibility to pathogens. Such immunosenescence has been described in a wide range of insects, including *Drosophila*, crickets, scorpion flies, and the mosquito *Ae. aegypti* [62], [63]. With respect to malaria vectors, while an early study suggested that the ability of *An. gambiae* to melanize foreign entities was reduced in old females [64], recent findings revealed no age effect on *An. gambiae* competence for fungi [65] or *P. falciparum* [44].

From the parasite perspective, although old mosquitoes might be more permissive hosts, they represent a major challenge for transmission. Since most mosquitoes do not live long enough to ensure the completion of parasite development, old vectors are expected to reduce future opportunities of transmission [66]. While some studies suggest that such time constraint can select parasites with shorter development duration (e.g., [67]), it is currently unknown whether malaria parasites can accelerate their sporogonic cycle when their transmission is compromised by the imminent death of their vector. Such condition-dependant developmental strategies, described in other parasite species [68], [69] and in blood-stage malaria parasites [70], deserve considerations in infected mosquitoes.

Despite extensive variation in body size within mosquito populations, its consequence on vector competence is still poorly documented. In general, it is often assumed that big animal hosts carry fewer parasites than small hosts because of higher investments in immunological defenses [71]. In contrast, when parasite development benefits from large nutritional resources, large hosts can suffer from high infection levels [72]. Further studies are needed to tease apart the relative importance of these two contrasting processes in malaria vectors where conflicting results have been reported. For example, one study observed that melanization of foreign objects was strongest in large females [28], while others found that smaller individuals were less likely to carry high number of oocysts, possibly due to difference in blood meal size during infection [73], [74].

### Other Overlooked Biotic and Abiotic Pressures

Accumulating evidence indicates that species interactions such as competition and predation can indirectly alter interactions with other community members, including parasites (trait-mediated indirect effect, [75]). For example, presence of predators can induce behavioral defenses in the preys, resulting in a change in susceptibility to parasites (e.g., [76], [77]). No studies to date have addressed the impact of these ecological parameters on mosquito vector-*Plasmodium* interactions. In *Aedes* mosquitoes however, studies showed that intra- and interspecific larval competition can increase vector competence for arboviruses [78]–[82]. These studies not only highlighted the importance of environmental factors experienced by immature stages with latent effects on competence in subsequent adult stages, they also revealed complex interactive effects of multiple environmental factors, including larval density, insecticide, and temperature [81]–[85].

## Evolutionary and Epidemiological Implications

**Figure 3:**
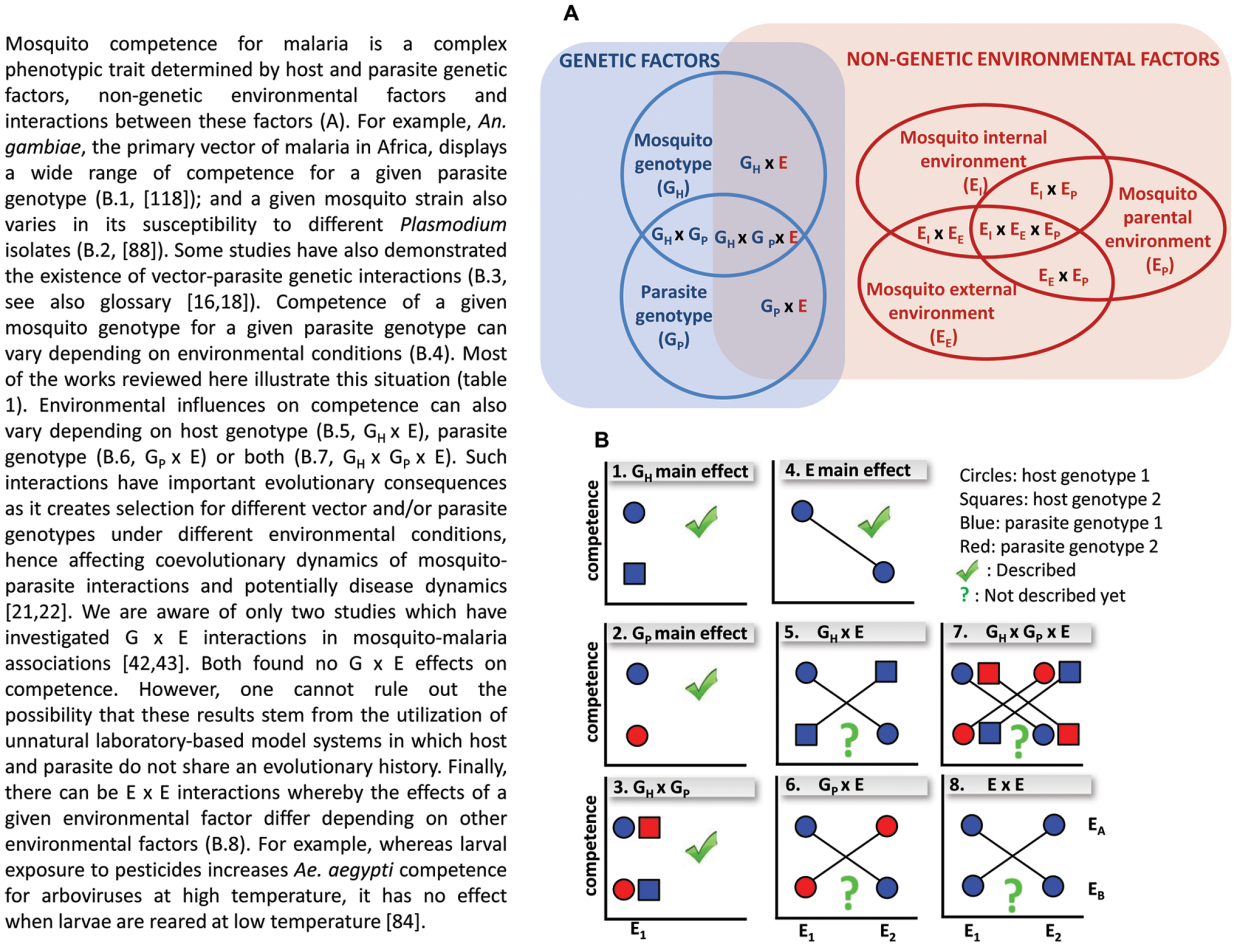
Disentangling the influence of host genotype, parasite genotype, environment, and interactions.

Taken together, the evidence reviewed here indicates that non-genetic factors can shape mosquito competence for malaria parasites; and this is likely to have profound evolutionary and epidemiological implications. One obvious implication is that the intensity of malaria transmission will vary spatially and temporally depending on biotic and abiotic environmental fluctuations. For example, the introduction of anti-*Plasmodium* gut bacteria in a vector population will decrease disease transmission. However, such simple predictions may be weakened by a number of complications. First, while our focus has been on competence, the various environmental parameters described here can also impact the other essential components of vectorial capacity, namely mosquito larval development, adult longevity, and biting rate, which all contribute to the overall dynamic of malaria. For example, some environmental parameters may decrease mosquito competence while increasing its longevity, thereby having no net effect or even enhancing malaria transmission. Second, the possibility exists that different environmental parameters have opposite, additive, or synergistic effects on competence (E×E interactions, **Figure 3**). For instance, temperatures at which malaria development is suppressed can depend on other factors such as humidity or mosquito diet and body size.

A primary evolutionary consequence of environmental variations is the maintenance of genetic diversity in both host and parasite populations, provided some genotypes are favored in one environment, whereas other genotypes perform better in other environments (i.e., G×E interactions with crossing reaction norms, **Figure 3**) [21], [22]. Although G×E effects on competence have not yet been described, G×E effects appear to be common in natural systems [22] and a study found *Plasmodium* genotype by diet interactions for mosquito survival [43]. Consistent with the existence of G×E effects, mosquito vectors and malaria parasites display extreme genetic polymorphism in immunity [86], [87] and infectivity [88], respectively. In turn, this can have important implications for conventional and genetic control strategies, since abundant polymorphism generally provides organisms with greater evolutionary capacity following environmental changes [86].

Some studies demonstrate that malaria infection is costly to the mosquito host. For example, *Plasmodium* infection can have negative impacts on vector reproduction and/or survival [89]–[92]. Furthermore, the insect's innate immune system reacts strongly to the parasite's presence [4]–[7], suggesting that the benefits of mounting costly physiological immune responses outweigh the cost of malaria infection. Since *Plasmodium* decreases mosquito fitness, natural selection should favor the evolution of defenses against it. Besides immunological defenses, insect hosts can use behavioral defenses including avoidance, behavioral fever, and self-medication to better resist or tolerate their parasites (**Figure 2**, [40], [93]–[95]). Despite increasing evidence for environment-mediated resistance against malaria parasites, mosquito behavioral immunity remains an overlooked defense strategy. We know of only one study that has explicitly tested for behavioral immunity in mosquitoes. Using a thermal gradient, this study investigated whether *P. yoelii*–infected *An. stephensi* could seek out warm resting sites that increase their body temperature to levels that are detrimental to malaria parasites [96]. Infected and uninfected individuals displayed similar temperature preference, therefore providing no support for the behavioral fever hypothesis. However, one cannot rule out the possibility that the absence of *Plasmodium* influence on mosquito thermal preference stemmed from the fact that the host-parasite association studied was unnatural [96].

Alternatively, malaria parasites might modify their insect host behavior in ways that favor their own development and transmission (**Figure 2**, [97]–[99]). Such parasite manipulation of vector behavior has been relatively well described in the context of mosquito blood-feeding, whereby sporozoites (mature, transmissible stages of malaria parasites) increase vector aggressiveness to favor between-hosts contact [98], [100]. Evidence for parasite exploitation of mosquito environment through behavioral manipulation is scarce; however, there is indirect evidence from a field study on *Plasmodium mexicanum*–infected sandflies [101]. Compared to their uninfected counterparts, infected sandflies were attracted by warmer temperatures, which were optimal for the parasite development rate but harmful to the development of sandfly eggs [101].

Regardless of whether hosts and/or parasites can exploit environmental conditions to their own benefit, non-genetic competence factors can have important consequences on the evolution of host physiological immunity and parasite virulence. First, because mounting an immune response is costly, environmental conditions that confer increased resistance will likely select for a reduced physiological immunity. For example, ants reduce the use of their immune system when antimicrobial resin is present in their nests [102]. On the other hand, environmental harnessing of parasite growth may select for increased virulence. Consistent with this prediction, theoretical and empirical studies showed that environmental factors such as antiparasitic host food or the presence of other parasite species or strains can select for more exploitative parasites [103]–[105].

Finally, environmental influences on mosquito competence can have contrasting evolutionary and epidemiological implications depending on whether mosquito resistance or tolerance is affected. Because resistance directly reduces parasite fitness, whereas tolerance does not (see **Glossary, File S1**), it is generally assumed that genetic polymorphisms in resistance, but not in tolerance, will be maintained; and that resistant hosts will decrease parasite transmission while tolerant hosts will increase it [106]–[108]. The reason for these differences is that resistance results in a negative epidemiological feedback where parasite infection selects for resistant hosts that will decrease parasite occurrence (assuming resistance is costly in the absence of disease, but its benefits outweigh the costs in the presence of disease). In contrast, tolerance evolution results in a positive feedback where parasite infection selects for tolerant hosts that will increase parasite occurrence. Likewise, environmental factors that confer tolerance will likely increase parasite transmission, while those conferring resistance will decrease it. For instance, if mosquitoes eat food that makes them healthier, they may live longer and transmit more parasites (tolerance), while if they eat food with antiparasitic properties, they would transmit less (resistance) (see [109] for a concrete example in butterflies). The study of mosquito defense mechanisms has typically focused on the ability to limit the infection [4]–[10]. However, growing evidence in vertebrate and insect hosts [110] highlights the need to turn our attention to mosquitoes' ability for damage control in addition to their ability for parasite control.

## Implications for Disease Surveillance and Control Strategies

In a time of renewed scientific and political commitment to malaria control, modeling the potential impact of environmental conditions on disease transmission is an obvious need. One strategy is to use environmental data to develop warning systems and risk maps that predict the incidence of malaria in our changing world. Until now, this approach has mostly focused on the effects of global warming, and it has elicited controversy in part because most of the climate-driven models assumed simple relationships between vectors, parasites, and temperature. Thus, if we are to accurately forecast the impact of environmental changes on malaria dynamics, we first need to recast malaria systems in their natural environment. As one concrete example, theoretical and empirical findings by Paaijimans et al. [37], [111] suggest that models neglecting diurnal temperature fluctuations will potentially overestimate malaria risk in warm regions but underestimate it in cooler regions (see also [112]).

Non-genetic determinants of competence can also contribute to the design of novel control strategies. For example, the introduction of non-native *Wolbachia* strains that suppress *Plasmodium* development has been proposed as a tangible method for malaria control [113], [114]. Alternatively, new potential strategies may take advantage of native parasites and gut bacteria, which have also been reported to reduce both sporogonic development and mosquito lifespan [51], [54], [56]. Although these approaches are promising, it is important to explore how other environmental parameters will interact with these potential biocontrol agents.

The possible release into natural mosquito populations of refractory transgenic mosquitoes has attracted considerable attention in recent years. A number of different genetic manipulation strategies are now available to reduce mosquito competence for *Plasmodium* in the laboratory [11], [12], [115]. However, before translating these findings into the natural settings, it will be crucial to determine how the transgenes expression will be affected by environmental variation [116], [117]. For example, the selected *Plasmodium*-refractory strain of *An. gambiae* [118] display varying melanization responses depending on temperature and larval diet [28]. There is no reason to believe that the competence of transgenic mosquitoes will not also be sensitive to non-genetic factors.

Finally, non-genetic-mediated competence can interfere with current control approaches in several ways. In particular, malaria control strategies relying on environmental modifications may have cascading effects on mosquito competence that may ultimately hinder the desired effects. Among other repercussions, the elimination of larval habitats can lead to intense competition in the remaining breeding sites and generate highly competent vectors [79]; insecticide control of malaria vectors can have indirect consequences on their competence for *Plasmodium* through pleiotropic effects of genetic changes (evolution of insecticide resistance) [119], and control measures targeting filarial worms may enhance malaria transmission in co-endemic areas [55].

## Outlook

Our intent has been to argue that non-genetic factors can strongly affect mosquito competence for malaria parasites and that evolutionary, epidemiological, and control implications can be profound. Despite early works on temperature effects and recent growing evidence, we are still far from a complete understanding of the environmental effects on mosquito-*Plasmodium* interactions. Compared to the effort devoted to the molecular and physiological study of mosquito immunology, relatively few studies have considered the ecological context in which mosquito immunity is expressed and has evolved. This is unfortunate, as it compromises our understanding of many aspects related to the evolution of the malaria vectorial system. This gap in our knowledge also limits both the implementation and development of sustainable control strategies. Thus, there is an urgent need to explore the effects of previously overlooked non-genetic factors (e.g., predation, competition), as well as reassess the effects of known factors such as temperature and diet by using ecologically relevant parameters (e.g., sympatric plant diversity instead of glucose solutions and fluctuating instead of constant temperatures). Furthermore, this research should ideally use natural vector-parasite combinations. Future studies should also determine whether or not generalizations are possible to address whether similar environmental conditions result in the same consequences for competence in a range of different mosquito-*Plasmodium* systems. Another major challenge will be to decipher the mechanisms by which non-genetic factors influence vector competence. In conclusion, considering the environmental context is not only crucial for understanding the outcome of mosquito-*Plasmodium* interactions but also for making more accurate predictions about the evolution of parasite transmission strategies and virulence, the evolution of mosquito immunity, and the dynamic of malaria transmission.

## Supporting Information

File S1 **Glossary of terms.**
(DOC)
