# Supplementary material for: Non-Genetic Determinants of Mosquito Competence for Malaria Parasites
Source: PLoS Pathog. 2013 Jun 20;9(6):e1003365. doi: 10.1371/journal.ppat.1003365 (PMC3688545; doi:10.1371/journal.ppat.1003365)
Supplement: File S1 — Glossary of terms. (DOC) [file ppat.1003365.s001.doc]

**Glossary:**

- **Host genotype by parasite genotype interactions** for competence occur when genetic variation within both host and parasite influence the outcome of infection such that mosquito genotypes that are competent for one parasite genotype are less competent for the others.

- **Host tolerance vs resistance**: host defensive mechanisms against parasites can be categorized into two types of strategy: resistance and tolerance. Resistance prevents infection, reduce parasite growth or clear infection, while tolerance alleviates the fitness costs from parasitism without limiting infection.

- **Immune priming** is a form of immunological memory whereby previous exposure to parasites stimulates the host immune system and results in protection on subsequent challenge by either the same or a different parasite strains or species. Increased immune protection following early exposure to parasites can occur in a single individual (within-generation priming) or in the next generation (trans-generational priming).

- **Melanization response**, or melanotic encapuslation response, is a form of innate immune response whereby an insect host produces and deposits melanin pigments on invading parasites to kill it.

- **Gut microbiota**: communities of naturally occurring microorganisms living in the mosquito gut.

- **Microsporidian parasite**: single celled, obligate, intracellular parasites with both horizontal and vertical transmission.

- **Trait-mediated indirect effects** occur when one species alters traits (e.g., behavioral, morphological) in a second species in ways that change the interaction between the second and third species.

- **Vectorial capacity (C)** is the number of infective bites received daily by a single host. C = (ma^2^VP^n^)/-ln P where m is the density of vectors in relation to density of vertebrate hosts; a the number of human bites per mosquito and per day, V the vector competence, P the daily survival of vectors and n the extrinsic incubation period (number of days required for sporogonic development)

***- Wolbachia*:** endosymbiotic bacteria with exclusively vertical transmission (maternal inheritance). *Wolbachia* are the most common parasitic microorganisms in insects and can manipulate the reproductive system of their hosts through feminization, males killing or cytoplasmic incompatibility.
